# Local-to-non-local transition laws for stiffness-tuneable monoatomic chains preserving springs mass

**DOI:** 10.1098/rsta.2024.0037

**Published:** 2024-08-12

**Authors:** Flavia Guarracino, Massimiliano Fraldi, Nicola M. Pugno

**Affiliations:** ^1^ Laboratory for Bioinspired, Bionic, Nano, Meta Materials and Mechanics, Department of Civil, Environmental and Mechanical Engineering, University of Trento, via Mesiano 77, Trento, Italy; ^2^ Department of Structures for Engineering and Architecture, University of Naples Federico II, via Claudio 21, Naples, Italy; ^3^ LPENS—Départment de Physique, Ecole Normale Supérieure, Paris, France; ^4^ School of Engineering and Materials Science, Queen Mary University of London, Mile End Road, London E1 4NS, UK

**Keywords:** nonlocal metamaterials, roton-like behaviour, mass conservation, wave propagation

## Abstract

Recently, non-local configurations have been proposed by adding beyond nearest neighbour couplings among elements in lattices to obtain roton-like dispersion relations and phase and group velocities with opposite signs. Even though the introduction of non-local elastic links in metamaterials has unlocked unprecedented possibilities, literature models and prototypes seem neither to provide criteria to compare local and non-local lattices nor to discuss any related rules governing the transition between the two configurations. A physically reasonable principle that monoatomic one-dimensional chains must obey to pass from single- to multi-connected systems is here proposed through a mass conservation law for elastic springs thereby introducing a suitable real dimensionless parameter α to tune stiffness distribution. Therefore, the dispersion relations as a function of α and of the *degree of non-locality* P are derived analytically, demonstrating that the proposed principle can be rather interpreted as a general mechanical consistency condition to preserve proper dynamics, involving the spring-to-bead mass ratio. Finally, after discussing qualitative results and deriving some useful inequalities, numerical simulations and two-dimensional FFTs are performed for some paradigmatic examples to highlight key dynamics features exhibited by chains with finite length as the parameters α and P vary.

This article is part of the theme issue ‘Current developments in elastic and acoustic metamaterials science (Part 2)’.

## 1. Introduction

Beyond nearest-neighbour (BNN) interactions are relevant in many fields of physics. In condensed matter physics, next-nearest-neighbour interactions have a significant influence on the material properties as is the case, for instance, with graphene, a two-dimensional material made of carbon atoms arranged in a hexagonal lattice, which leads to non-local electronic hoppings [1]. The influence of long-range couplings on the dispersion characteristics and dynamical response of two-dimensional and three-dimensional lattices is considerable, as the strength of interaction forces impacts wave and energy propagation [2–4].

In electromagnetism, the concept has paved the way to potential waveguiding applications, especially in optics, that is in the visible range of electromagnetic wave frequencies [5–8]. Dynamical processes in classical mechanics can be influenced by BNN interactions as well. Their counterparts in statics have been extensively studied in the past, and both continuous [9,10] and discrete [11] models have been analysed since the first contributions. Non-local elastic models in continua discussed in classical literature works, as in the works by Eringen *et al*. [9,12,13], present the challenge of adapting the constitutive stress–strain relationship of non-local classes of materials, so that their constitutive response be attenuated with the distance between two arbitrarily distant points. For this purpose, the authors introduced an influence function with an exponential law to ensure the decay of the elastic moduli along with the distance between the given points. More recently, alternative non-local models have been proposed for elastic and fracturing non-local metamaterials. Peridynamic models are among these, as the bond–bond ones proposed by Silling [14], where a power law is employed with the aim of decreasing the links’ stiffness when farther from the so-called peridynamic horizon.

A few real materials are known to exhibit a non-local microstructure. A two-dimensional existing man-made material with evidence of non-local organization is constituted by some types of recycled or cold-pressed sheets of paper, in which connections and entanglements of intricate networks of linear elastic filaments can be recognized at a microscopic level and influence their fracture mechanisms.

**Figure 1.**
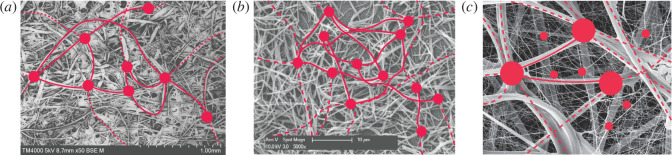
In this figure, three materials exhibiting a non-local microstructure are shown through pictures taken by SEM, and adapted to highlight the non-local links. In (*a*) a scan of rice-paper is shown in a capture performed at the LIMiTS laboratory of the University of Naples Federico II with SEM Hitachi series TM4000 plus. The rice-paper is composed by cold-pressed sheets with several non-local links. In (*b*) a SEM scan from Kim *et al*. [15] is adapted, where polydioctylfluorene nanorods are interlaced and non-locally paired. Finally, in (*c*) a SEM image of spider silk from https://www.sciencephoto.com/media/373522/view/spider-s-web-sem is overlapped by a scheme that highlights the multiple, hierarchical non-local connections.

Non-local pairings can furthermore be observed in nanorods of different elements [15], in spider silk and in polymer entanglements. Due to their non-locality, these materials have encouraged the development of several non-local models in statics and dynamics, both in the continuum and discrete domain through different approaches, from constitutive boundary conditions to peridynamics, to fractional models [16–19]. From figure 1, the non-local sub-structure of some of the materials cited above can be observed through scanning electron microscope (SEM) images, on which a possible simplified scheme of the pairings has been illustrated.

In recent years, the topic has gained the attention of the researchers due to its potential breeding with metamaterial design and thus its role in wave propagation, localization or energy harvesting. In fact, tunability and shifts in the frequency range are of utmost importance in metamaterial devices, as they encompass a broad set of scales and applications [20,21], and BNN connections stretch the boundaries for their design. The seminal work by Brillouin [22] already takes into account non-local couplings in the theory of wave propagation in monoatomic lattices, and provides the dispersion relation for a set number of BNN couplings as early as in 1956. In the phononic crystals and metamaterials design field, the influence of BNN interactions can be of crucial importance in governing waves and thus energy propagation. As periodic structures, they can be analysed through Bloch’s theory, originally applied to crystalline structures [23], the overview of their dynamical behaviour can be read on a dispersion curve. Moreover, some studies were carried out to analyse the interaction forces in a non-local infinite periodic chain [24] and the response to the motion of one element [25]. The potential of non-locally connected lattices in applications is tied to the resulting ‘roton-like’ dispersion relation, which is peculiar because it has a curve presenting a local minimum, corresponding to an inversion of sign of the group velocity (velocity at which the wave packet and thus the energy moves, as opposed to phase velocity which corresponds to the velocity at which the wave front moves forward). This concept, which might seem fairly new in mechanics, has however a long history: in 1962, Landau was awarded the Nobel prize for identifying what he named ‘rotons’ in the dispersion relations of superfluid 4He at low temperatures [26], that were later detected by Feynman in experiments on inelastic neutron scattering in 1961 [27]. The rotons are also observed in Bose–Einstein condensates, and they still represent an active research branch [28–30].

In recent years, some studies [31,32] have proven it possible to obtain the same kind of dispersion diagram experimentally in *ad hoc* designed phononic crystals and periodic metamaterials. In this case, there is no contribution of quantum effects involved and no correlation to low temperatures, to great advantage of experimental stages. Possible applications can even mingle with other relevant topics in the metamaterials community, as topology and disordered lattices [33,34], where they contribute to the appearance of localized modes. In mechanics, increasing the BNN interactions causes the number of local minima (corresponding to rotons) and maxima (corresponding to maxons) to increase accordingly [35,36]. Taking advantage of this feature to obtain specific dispersion curves, a study by Kazemi *et al*. [36] produced a detailed method for tailoring specific dispersion relations through the usage of Fourier series coefficients as spring constants, proving how powerful a tool BNN interactions in metamaterials can be and employing some parameters to design curves fitting to certain goals of wave propagation and zero group velocity modes. In [37], Sepehri *et al*. examine the behaviour of the non-local phononic crystals when the nonlinearity comes into play. A monoatomic chain with third BNN interactions is the reference model with roton-like dispersion relation and backwards wave propagation as a result. They introduce nonlinearity as a means of studying amplitude-dependent behaviour, which could enhance the capacity for tunability.

Interestingly, the roton-like behaviour in dispersion curves was proven not to be solely tied to BNN connections, but also to hybridization and/or polarization of modes in elastic, electromagnetic and acoustic waves [38–42].

Martìnez *et al*. [43] provided experimental proof of the roton-like dispersion relations in three-dimensional metamaterials through BNN. Two different experiments were carried out, one with transverse elastic waves in microscale materials and ultrasound frequencies, the other with longitudinal air-pressure waves in macroscopic channel-based materials at audible frequencies. They underlined how the real advantage of the BNN interactions is in tailoring the lower branch while the other metamaterial studies focused on tweaking higher branches. In [44], BNN is used to design a metamaterial with backwards propagating waves, i.e. with different signs in group and phase velocity. The numerical and experimental model is three-dimensional, achieved through Meccano construction elements, which gives rise to a prototype with interchangeable pieces. A paper by Zhu *et al*. [45] focuses on multidirectional roton-like dispersion relations following the work from Chen *et al*. [31], by developing an experiment on roton-like dispersion relations in acoustical metamaterial at ambient temperature. In this manner backwards waves are obtained, in analogy to the return flow defined by Feynman [27].

A sound mechanical and physical foundation of the way in which the non-localities are devised is however generally missing in the majority of these models. In fact, a simple increase in couplings implies a variation in the overall stiffness and different scaling laws do allow for a straightforward physical comparison between different models, being local or non-local. Most importantly, a paradox could arise as most of the studies cited above perform their preliminary analyses on a mass-spring model. In fact, the main assumption in employing these simplified models is that the only masses that participate in the dynamics of the system are those of the atoms, while the springs’ masses are considered negligible. However, when the number of non-local pairings increases, the mass of the springs increases accordingly, and disregarding it in the dynamical analyses could lead to the model’s inconsistency. An example of this contradictory case can be for instance imagined for a system as the one studied and actually manufactured as a prototype by Park *et al*. [46]. If one added an arbitrary number of non-local springs to the flexural metamaterial model without preserving spring mass, the same springs’ masses would rapidly become of the order of magnitude of the oscillating masses as the number of elastic connections increases: as a result, that material would be called into play participating in the dynamics, in clear contrast to the hypotheses that consider the springs’ mass negligible.

The present study explores the various implications of enforcing additional BNN couplings in a scenario where the amount of material is limited, and an order of magnitude ratio is enforced between the springs’ and the atoms’ masses. Thus, a novel mass conservation law is proposed, in which a stiffness distribution parameter α rules the non-locality of choice of the system and the dispersion characteristics. In this manner, a rational comparison can be made between local and non-local models, on the basis of a different distribution and arrangement of the same amount of material, which is a common occurrence in designing any structural component and may be thus reasonably applied to metamaterials.

With the aim of gaining some insights into how non-local elasticity might contribute to suggest new optimization strategies for designing one-dimensional structures and possibly three-dimensional lattices and metamaterials, the work makes reference to the simplest paradigm of a mono-atomic chain made of masses equally distanced between each other and mutually connected by linearly elastic springs. Dispersion relations for such one-dimensional systems are then obtained and the dynamic response of selected case studies is analysed by forcing the mass (or equivalently the volume) of the elastic links branching off from each single mass to be constant.

This article is structured as follows: in §1, a basic overview of the physical principles and mathematical methods behind the analysis of a non-local one-dimensional monoatomic chain is provided; in §2, the proposed mass-conservation constraints are introduced and a numerical simulation of the behaviour of the model in a finite chain scenario is presented, thereafter, the consequences of the introduced assumptions are investigated; in §3, the advantages and limitations of this approach are finally highlighted and discussed.

## 2. Modelling and analysis of non-local monoatomic chains

In this section, the proposed material constraint is presented, preceded by a swift summary of the mathematical tools employed in the study and their physical meaning. The implications of the adopted approach are then unfolded both through the theoretical framework cited above and explicit dynamics simulations.

### (a) Fundamentals on Bloch–Floquet monoatomic chains equipped with non-local springs

**Figure 2.**
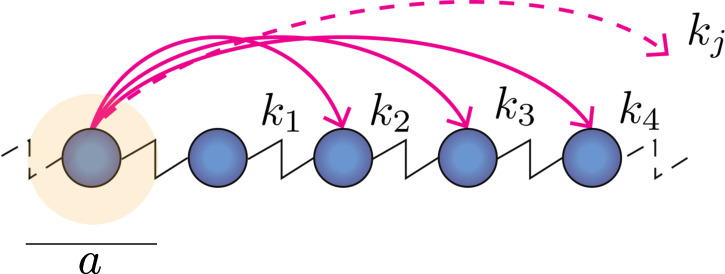
Scheme of the periodic non-local one-dimensional mass-spring chain. The highlighted area represents the unit cell, of length a, with its P number of connections with stiffness $k_{1},k_{2}...k_{j}$ to the other unit cells at distance aj, which feature the same connections to the neighbours.

The classical one-dimensional model of a monoatomic chain made out of an arbitrary number N of masses and (N−1) springs is considered. The system is assumed periodic with Bloch–Floquet boundary conditions, with a unit cell repeating itself at the lattice length a. If the generic mono-particle unit cell is elastically linked to the adjacent units as well as to distant masses through additional spring connections, a non-local version of the monoatomic chain—the BNN model outlined in figure 2—is obtained, whose mathematical formulation was first introduced in the classical book *Wave propagation in Periodic Structures* by Brillouin [22].

To characterize such dynamic systems, the energy–momentum dispersion relation—written in terms of frequency against wavenumber—has to be first derived. Exactly as in the standard case of local chains, by writing the equilibrium through Newton’s second law for the generic *n*th mass and neglecting viscous contributions, one reads


(2.1) $$F_{n}=m{\ddot{x}}_{n}=\sum_{j=1}^{P}k_{j}(x_{n-j}-2x_{n}+x_{n+j}),\;\;\;\;\forall n\in \{1,2,...,N-1,N\}\subset N,\;\;P|\{P<N,\;P\in N\},$$

where $x_{n}$ is the actual position of the *n*th mass and $k_{j}$ is the stiffness of the spring connecting two masses distanced by j−1 elements (i.e. j=1 denotes local springs and 1<j≤P refers to non-local connections).

The solution to this equation is then obtained through Bloch–Floquet theory for periodic systems, which assumes a travelling wave solution with a phase factor as follows:


(2.2) $$x_{n}=Ce^{i\omega t-i\kappa x_{n}^{eq}}=Ce^{i\omega t-i\kappa na},$$

(2.3) $$x_{n-j}=Ce^{i\omega t-i\kappa x_{n-j}^{eq}}=Ce^{i\omega t-i\kappa (n-j)a},$$

(2.4) $$x_{n+j}=Ce^{i\omega t-i\kappa x_{n+j}^{eq}}=Ce^{i\omega t-i\kappa (n+j)a},$$

where the apex eq refers to equilibrium position of the masses, *C* is an arbitrary constant representing the displacement amplitude, a is the unit cell length, κ is the wavenumber, the exponential form $e^{i\omega t-i\kappa x_{n}^{eq}}$ is the travelling wave and the periodicity indicates that the solution moving away from the unit cell varies by a factor $e^{i\kappa ja}$.

Inserting the solution into the equilibrium equation yields:


(2.5) $$C[2\sum_{j=1}^{P}(k_{j}-k_{j}\cos j\kappa a-m\omega^{2})]=0,\;\;\;\forall C\in R,$$

so that, after some algebraic manipulations, the dispersion relation is given by


(2.6) $$\omega =\omega (\kappa )=\sqrt{2m^{-1}\sum_{j=1}^{P}k_{j}(1-cos\left(j\kappa a)\right)}=\frac{2}{\sqrt{m}}\sqrt{\sum_{j=1}^{P}k_{j}sin^{2}(j\kappa \frac{a}{2})},$$

where the wavenumber κ is defined in the interval [−π/a,π/a], corresponding to the length of the 1st Brillouin zone in the so-called reciprocal space or lattice, while it is worth to notice that the stiffnesses $k_{j}$ are at this stage not yet explicitly given.

Key quantities like non-local monoatomic chain phase and group velocities can also be obtained. In fact, the dispersion relation provides information on the relationship between the angular frequency of the waves ω and their wavenumber κ. In particular, the group velocity—the velocity at which the envelope of wave packets travels—is then defined as the derivative of the dispersion relation over κ, thus representing the slope of the above-mentioned ω−κ graph and, in the present case, it takes the form:


(2.7) $$v_{gr}=\frac{\partial \omega (\kappa )}{\partial \kappa }=\frac{a}{2\sqrt{m}}\frac{\sum_{j=1}^{P}jk_{j}\sin\left(j\kappa a\right)}{\sqrt{\sum_{j=1}^{P}k_{j}\sin\left(j\kappa \frac{a}{2}\right)}}.$$

As it is well-known, in such dynamical systems, it is particularly important to compare the sign of the group velocity with that of the phase velocity $v_{ph}$, defined instead as the ratio of ω to κ, which in the case at hand takes the form:


(2.8) $$v_{ph}=\frac{\omega (\kappa )}{\kappa }=\frac{2}{\kappa \sqrt{m}}\sqrt{\sum_{j=1}^{P}k_{j}\;sin^{2}(j\kappa \frac{a}{2})},$$

which describes the rate of propagation of a component of the wave packet. When the ratio between the group and the phase velocity is negative, backwards-propagating waves take place, while instead when the group velocity is zero, zero-group velocity modes appear and no energy is propagated through the lattice, both cases being fundamental, for example, for characterizing dynamics of metamaterials.

It is worth noticing that, from the equations above, the study of the sign of the ratio of group and phase velocities can be helpfully reduced to analyse the following function sign


(2.9) $$s\equiv sgn[\frac{v_{gr}}{v_{ph}}]\propto \sum_{j=1}^{P}jk_{j}\sin\left(j\kappa a\right),$$

limiting to consider its behaviour in the symmetrical Brillouin hemi-region κ∈[−π/a,π/a], once the explicit expression for stiffness $k_{j}$ is provided.

### (b) The proposed *mechanical consistency condition* to govern local-to-non-local transition in BNN chains

The key idea of the present study is to set a framework to build BNN systems obeying the previous governing equations and characterized by various degrees of non-locality P, by prescribing necessary rules on the ratio between mass of material to be used, respectively, for the elastic springs and the atoms. This allows us first of all to ensure mechanical consistency of the model when a large amount of non-local pairings are entailed and to compare and evaluate optimal configurations for different non-local chains, paving the way to establish design criteria and to envisage new paradigms for conceiving non-local metamaterials.

In order to do so, under the hypothesis of periodicity of the chain system, the proposed constraint keeps the total mass of the links converging in a given atom constant, no matter how many BNN pairings are involved. In particular, the mass of each bead m is assumed constant, while the mass of the spring can be individually variable. In doing so, the inertia of the links, radiating out from that bead, must be regarded as negligible in order not to contribute to the dynamics of the chain. For this reason, a conservation law is here imposed to the total springs’ mass $m_{s}$. Moreover, since density of the spring material $\rho_{s}$ is considered constant, the proposed scaling can be consequently applied only to the total volume $V_{s}$ of the elastic links afferent to the periodic unit cell, centred on the *n*th atom. In addition, the length of every spring is considered as given by the distance between the two masses it connects, with a being the minimum length. Hence the proposed scaling law involves only the cross-section of the links.

Assuming a certain value $V_{s}=V_{const}$ for the volume, and considering different levels of connection in the infinite chain, the volume balance of the unit cell of a chain with a P level of non-locality reads


(2.10) $$\sum_{j=1}^{P}A_{j}^{\left(P\right)}ja=V_{const},$$

so that whatever is the level of connection and, consequently, the number of link cross-sections to be considered in the equation, the overall volume of the springs, i.e. the amount of mass, remains constant.

#### (i) Hypothesis of power law for scaling elastic spring stiffness with distance

Once the conservation of mass for the elastic links is imposed, how springs stiffness scales with the inter-atomic distances remains unconstrained because the conservation equation, imposed in terms of constant volume $V_{const}$, involves P geometric quantities, the cross-sectional areas $A_{j}^{\left(P\right)}$. To constrain these geometric parameters and to be able to solve the equation, a new equation is introduced. It assumes a sufficiently general power law for describing how stiffness had to scale with distances, with a stiffness distribution parameter α spanning over both positive and negative values. The condition is


(2.11) $$A_{j}^{\left(P\right)}=A_{1}^{\left(P\right)}j^{-\alpha },\;\;\;\alpha \in R,$$

where the exponential α is introduced as a *stiffness distribution* parameter imposed beforehand that will serve the purpose of increasing or decreasing the stiffness on a *j*th non-local spring. This choice adds a second parameter to the model, so that setting both the level of non-locality P and the stiffness distribution parameter α is required in order to control the model itself.

Substituting equation (2.11) in the volume conservation equation (2.10), for a generalized *P*th next nearest interacting chain the cross-section of the local links is then defined through the α parameter as follows:


(2.12) $$A_{1}^{\left(P\right)}=\frac{V_{const}}{a\sum_{j=1}^{P}j^{1-\alpha }}\cdot$$

The stiffness of the springs is defined as


(2.13) $$k_{j}^{\left(P\right)}=\frac{EA_{j}^{\left(P\right)}}{a_{j}},$$

where E is the Young modulus of the spring material, $a_{j}$ is the length of the *j*th link, which equals to ja, and $A_{j}^{\left(P\right)}$ is the corresponding cross-section, in which the subscript indicates it connects the *j*th next neighbour and the superscript denotes the maximum number of BNN connections in the designed chain. In fact, for a mono-atomic (discrete) model of masses and springs with linear (local or non-local) and elastic interactions, every mass has one degree of freedom along the axis of the system and, therefore, the simplest way to connect elastically (nearest or beyond next nearest) masses is to assume linearly elastic prismatic bars with cross-section area A and length L, from which the equivalent spring stiffness results as EA/L, where E is the Young modulus of the material.

$k_{j}^{\left(P\right)}$ can be defined as follows, from equations (2.11)−(2.13):


(2.14) $$k_{j}^{\left(P\right)}=\frac{A_{j}^{\left(P\right)}E}{a_{j}}=\frac{E}{a_{j}}\frac{A_{1}^{\left(P\right)}}{j^{\alpha }}=\frac{EV_{cost}}{a^{2}(j^{1+\alpha })\sum_{j=1}^{P}j^{1-\alpha }},\;\;a_{j}=ja,\;\;\;j\in \{1,...,P\}.$$

In equation (2.14), the stiffness distribution following the index of the BNN connected by the *j*th spring and α is clear, as the sole non-constant value, once the values of $E,V_{cost},a,P$ and α for the chain are set, is $\left(j^{1+\alpha }\right)$. Depending of the non-locality level of the spring, its stiffness will depend on the α value selected. As α>0, the classical hyperbolic scaling law of stiffness with distance is recovered, while as α<0, unconventional albeit possible stiffness values that increase with inter-atomic distances are obtained.

**Figure 3.**
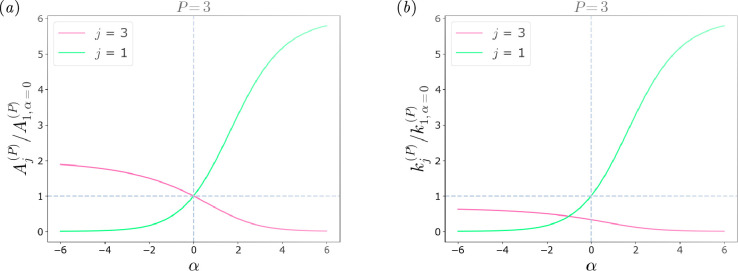
Considering a monoatomic chain with a level of non-locality P=3, the graphs show how (*a*) $A_{j}^{\left(P\right)}$ and (*b*) $k_{j}^{\left(P\right)}$ vary for different α values in the most local and non-local springs. The values are normalized, respectively, over $A_{1}^{\left(P\right)}$ and $k_{1}^{\left(P\right)}$ calculated for α=0.

Examples of the ratio of $A_{j}^{\left(P\right)}/A_{1,\alpha =0}^{\left(P\right)}$ and $k_{j}^{\left(P\right)}/k_{1,\alpha =0}^{\left(P\right)}$ are presented in figure 3. By varying the parameter α within the range of α∈[−6,6], the effects on the cross-section (figure 3*a*) and on the stiffness (figure 3*b*) of the $P_{th}$ next neighbour are observed. The green curves represent the area and the stiffness of the spring connecting the third BNN neighbour, and they have opposite concavities compared with the pink curves, which refer to the local connections. When the value of α increases, the cross-section and the stiffness of the local links increase accordingly, while the non-local counterparts decrease. The opposite behaviour is observed when α assumes negative values.

#### (ii) Explicit dispersion relation, group and phase velocities

Once the mass conservation for springs is imposed and how elastic spring stiffness scales with distance is defined, some relevant quantities for describing the dynamics of non-local mono-atomic chains can be explicitly given.

In particular, from (2.6), the dispersion relation follows:


(2.15) $$\omega =2\sqrt{\frac{EV_{const}}{ma^{2}}}\sqrt{(\sum_{j=1}^{P}j^{1-\alpha }{)}^{-1}\sum_{j=1}^{P}j^{-(1+\alpha )}\sin^{2}(j\kappa \frac{a}{2})}.$$

Recalling the above-mentioned discussion on the *mechanical consistency condition* and its relation with the admissibility of preserving arbitrary low spring-to-bead mass ratios, it is helpful to introduce this masses ratio explicitly as:


(2.16) $$\mu =\frac{m_{s}}{m}=\frac{\rho_{s}V_{s}}{m}\equiv \frac{\rho_{s}V_{const}}{m}\ll 1,$$

which ensures that mono-atomic chain dynamics involves the oscillating atoms only, the inertial contribution of spring mass being negligible regardless of the number of elastic links and hence of the level of non-locality P. Recalling that elastic waves in the spring material travel at the speed $v_{S}$, it follows:


(2.17) $$v_{s}=\sqrt{\frac{E}{m_{s}}}\;⟹\;V_{const}=\frac{\mu mv_{s}^{2}}{E},$$

from which the final dispersion relation can be effectively rewritten as


(2.18) $$\omega =\frac{2\sqrt{\mu }}{\tau }\sqrt{(\sum_{j=1}^{P}j^{1-\alpha }{)}^{-1}\sum_{j=1}^{P}j^{-(1+\alpha )}\sin^{2}(j\kappa \frac{a}{2})},\;\;\;\tau =\frac{a}{v_{s}}>0,$$

τ being a *characteristic time* of the system, representing the time spent by the elastic wave to travel inside the spring material to cover the minimum distance a between two near-chain atoms. As the mass ratio μ, τ also plays a crucial role in describing the actual dynamics of such one-dimensional systems, since it tells us about possible scattering or interference phenomena resulting from the competition between group and phase velocities and intrinsic (spring material) velocity $v_{s}$ of elastic waves, running along the links of the chain non-local network.

This is why group and phase velocities are specialized to the case in which the stiffness coefficients $k_{j}$ obey the conservation of mass (i.e. equation (2.14)) and are, respectively rewritten below as a function of the parameters μ and τ as well


(2.19) $$v_{gr}=\frac{\sqrt{\mu }}{2\tau }\frac{\sqrt{{\left(\sum_{j=1}^{P}j^{1-\alpha }\right)}^{-1}}\sum_{j=1}^{P}\sin(j\kappa a)j^{-\alpha }}{\sqrt{\sum_{j=1}^{P}j^{-(1+\alpha )}\sin^{2}(j\kappa \frac{a}{2})}},$$

and


(2.20) $$v_{ph}=\frac{2\sqrt{\mu }}{\tau \kappa }\sqrt{(\sum_{j=1}^{P}j^{1-\alpha }{)}^{-1}\sum_{j=1}^{P}j^{-(1+\alpha )}\sin^{2}(j\kappa \frac{a}{2})}.$$

Importantly, by means of some algebraic manipulations, it follows that the sign function can now be explicitly estimated as


(2.21) $$s\equiv sgn[\frac{v_{gr}}{v_{ph}}]\propto \sum_{j=1}^{P}j^{-\alpha }\sin\left(j\beta \pi \right),\;\;\kappa =\frac{\beta \pi }{a},\;\;\beta \in (0,1),$$

where β was introduced to make reference to a dimensionless sign function to be studied in the symmetrical Brillouin zone of positive wavenumbers κ.

### (c) Some useful inequalities for non-local mono-atomic chains

The formulas obtained above reveal some remarkable qualitative results.

One emerges from the analysis of the sign function (2.21), which plotted for β∈[0,1] and arbitrary integers P shows that the group and phase velocities are always coherent, provided that α is greater than or equal to one, that is


(2.22) $$\forall \alpha \geq 1,\;\;s\equiv sgn[\frac{v_{gr}}{v_{ph}}]\geq 0,\;\;\;\{\forall \beta \in [0,1]\subset R^{+},\;\;\forall P\in [1,+\infty [\subseteq N\}.$$

Within the framework of metamaterials to be designed for exhibiting opposite-in-sign group and phase velocities, this inequality gives key information stating that, regardless of the grade of non-locality P, mono-atomic chains whose masses are connected with elastic springs that scale as in (2.14) can dynamically retrieve the roton-like behaviour if and only if the stiffness tuning parameter α<1.

Also, with in mind metamaterials exhibiting band gap, some universal as well as α-dependent upper bounds for frequencies ω can be found in the Brillouin zone from the obtained non-local dispersion relation.

A first general upper bound for ω can be derived taking advantage of the mathematical terms of the sums as follows:


(2.23) $$\frac{\tau^{2}}{4\mu }\omega^{2}=\frac{\sum_{j=1}^{P}j^{-(1+\alpha )}\sin^{2}(j\kappa \frac{a}{2})}{\sum_{j=1}^{P}j^{1-\alpha }}\leq \frac{\sum_{j=1}^{P}j^{-(1+\alpha )}}{\sum_{j=1}^{P}j^{1-\alpha }}\leq \frac{\sum_{j=1}^{P}j^{1-\alpha }j^{-2}}{\sum_{j=1}^{P}j^{1-\alpha }}\leq 1\Rightarrow \omega \leq \frac{2\sqrt{\mu }}{\tau },\;\forall \alpha \in R,$$

from which no waves at frequencies over the obtained upper bound can travel in the mono-atomic chain, regardless of the level of non-locality P and independently from the way in which the stiffness of non-local springs is tuned with distance through the parameter α.

Another better upper bound for ω, finally given analytically as a function of α and P, can be found by exploiting the Cauchy–Schwarz inequality, but for α≥1. In fact, being all coefficients in the sums non-negative terms, one first writes:


(2.24) $$\frac{\tau^{2}}{4\mu }\omega^{2}=\frac{\sum_{j=1}^{P}c_{j}(\alpha )j^{-2}s_{j}^{2}}{\sum_{j=1}^{P}c_{j}(\alpha )}\leq \frac{(\sum_{j=1}^{P}c_{j}(\alpha {)}^{2}j^{-4}{)}^{\frac{1}{2}}(\sum_{j=1}^{P}s_{j}^{4}{)}^{\frac{1}{2}}}{\sum_{j=1}^{P}c_{j}(\alpha )}\leq \frac{(\sum_{j=1}^{P}c_{j}(\alpha {)}^{2}{)}^{\frac{1}{2}}(\sum_{j=1}^{P}s_{j}^{4}{)}^{\frac{1}{2}}}{\sum_{j=1}^{P}c_{j}(\alpha )},\;\forall \alpha \in R,$$

where, for simplicity, we baptized $c_{j}$ and $s_{j}(\alpha )$ as follows


(2.25) $$c_{j}(\alpha )=j^{1-\alpha },\;\;\;\;s_{j}=\sin(j\kappa \frac{a}{2}).$$

By noticing that


(2.26) $$\sum_{j=1}^{P}c_{j}(\alpha {)}^{2}\geq 1\Rightarrow (\sum_{j=1}^{P}c_{j}(\alpha {)}^{2}{)}^{\frac{1}{2}}<\sum_{j=1}^{P}c_{j}(\alpha {)}^{2},\;\;\;\forall \alpha \in R,$$

it is possible to continue the chain of inequalities as follows


(2.27) $$\frac{\tau^{2}}{4\mu }\omega^{2}=\frac{\sum_{j=1}^{P}c_{j}(\alpha )j^{-2}s_{j}^{2}}{\sum_{j=1}^{P}c_{j}(\alpha )}\leq \;\frac{(\sum_{j=1}^{P}c_{j}(\alpha {)}^{2})(\sum_{j=1}^{P}s_{j}^{4}{)}^{\frac{1}{2}}}{\sum_{j=1}^{P}c_{j}(\alpha )},\;\;\;\forall \alpha \in R.$$

Finally, if α≥1, one has


(2.28) $$\frac{\tau^{2}}{4\mu }\omega^{2}=\frac{\sum_{j=1}^{P}c_{j}(\alpha )j^{-2}s_{j}^{2}}{\sum_{j=1}^{P}c_{j}(\alpha )}\leq \;(\sum_{j=1}^{P}s_{j}^{4}{)}^{\frac{1}{2}}=(\sum_{j=1}^{P}\sin^{4}(j\kappa \frac{a}{2}){)}^{\frac{1}{2}},\;\;\;\forall \alpha \geq 1,$$

from which, since the sum at the right hand has analytical expression, one has


(2.29) $$\omega \leq \frac{\sqrt{\mu }}{\tau }\sqrt[{4}]{\frac{\sin(a\kappa (2P+1))}{\sin(a\kappa )}-\frac{4\sin(\frac{a\kappa (2P+1)}{2})}{\sin(\frac{a\kappa }{2})}+6P+3},\;\;\;\forall \alpha \geq 1,$$

which provides a new upper bound for the frequencies related to non-local mono-atomic chains.

### (d) Effects of the stiffness tuning parameter α on the model

**Figure 4.**
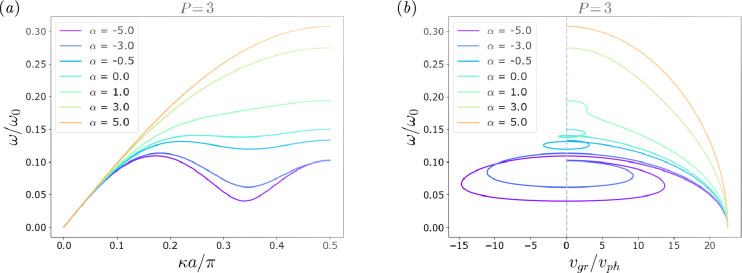
Considering a chain with one local and two non-local pairings (P=3), in (*a*) the dispersion curves for different values of α can be observed and in (*b*) the corresponding group velocities $v_{g}$ normalized over the phase velocity $v_{ph}$. The plot entails $\frac{\omega (\kappa )}{\omega_{0}}$ where $\omega_{0}$ is $v_{ph}/a$ versus $\frac{v_{gr}}{v_{ph}}$, where $v_{gr}$ is the group velocity over $v_{ph}$, the phase velocity. The graph in (*a*) shows the dispersion curves for the chain with P=3 for α∈[−5,5]. Each curve corresponds to a value of α, as stated in the legend. For positive values of α, it can be noticed how the plots appear as classical curves for a discrete local mass-spring chain, while for negative values of alpha the local minimum corresponding to the roton-like curve in non-local chains can be observed. Following the same reasoning, in (*b*) the vertical line in $\frac{v_{gr}}{v_{ph}}$ separates the positive from the negative values of the group velocity (as the phase velocity is always positive for positive wavenumbers). For negative values of α a larger set of negative $\frac{v_{gr}}{v_{ph}}$ values is attained.

A graphical example of the impact of the two design parameters α and P is described in figure 4*a,b*, where the dispersion curves and the group velocity values for a monoatomic chain with a level of non-locality P=3 are shown for different values of α. Even though the level of non-locality is fixed, the dynamic behaviour of the chain varies greatly based on how the stiffness is distributed among the links, as it can be observed from the legend, through the assignation of a value of α∈[−5,5]. The chains with negative α values exhibit a roton-like behaviour with a local minimum (figure 4*a*) and negative group velocity (figure 4*b*), while the non-local chains with stiffness decreasing with non-locality present a curve similar to that of a local monoatomic chain.

Once the general expressions are obtained, the dispersion curves for different values of α can be derived and the effect on the dynamical behaviour of the chain can be observed, some physically relevant cases emerging from the variation of this stiffness tuning parameter.

In particular, for α=0, it is found from equation (2.12) that $A_{j}^{\left(P\right)}$ becomes constant and loses its dependency on j, the non-locality of the spring in question:


(2.30) $$A_{j}^{\left(P\right)}=\frac{A_{1}^{\left(P\right)}}{j^{0}}=A_{1}^{\left(P\right)}=A^{\left(P\right)}.$$

In this case, it is $A_{j}^{\left(P\right)}=A^{\left(P\right)}$ and thus the stiffness of the links results proportional to the inverse of the lengths and to the degree of connection


(2.31) $$k_{j}^{\left(P\right)}\propto (ja{)}^{-1}\propto j^{-1},$$

hence the stiffness of the link decays as the distance from the connected neighbour increases.

On the other hand, for α=−1, $k_{j}^{\left(P\right)}$ becomes constant and can be expressed in analytical form,


(2.32) $$k_{j}^{\left(P\right)}=\frac{EV_{const}}{a^{2}j^{0}\sum_{j=1}^{P}j^{2}}=\frac{6EV_{const}}{a^{2}P(P+1)(2P+1)}\equiv k^{\left(P\right)},\;\;\;\forall P.$$

This means that every spring has the same stiffness regardless of the enforced degree of non-locality.

**Figure 5.**
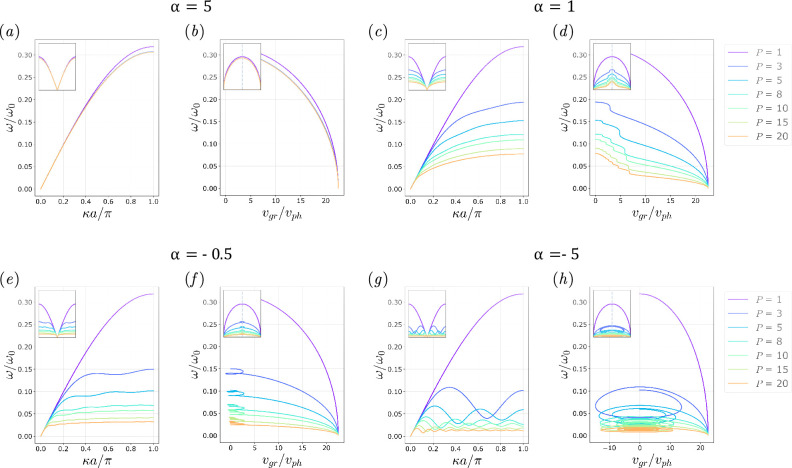
In the figure dispersion relations for irreducible Brillouin zones κ=[0,π/a] and the corresponding group velocities are shown for different degrees of non-local pairings in the monoatomic chain (*a*),(*b*) for α=5, (*c*),(*d*) for α=1, (*e*),(*f*) for α=−0.5 and (*g*),(*h*) for α=−5. It is evident that in the case α=5, the behaviour of the chain is strongly local, be it connected with one spring or many, while for the other limiting case, α=−5, several minima and maxima (rotons and maxons) are clearly visible in the curves with P>1 and consequently inversions in the sign of the group over phase velocity ratio can be observed. Noteworthily, in the case α=−0.5, the group velocity reaches an inversion in sign, however, for a limited amount of values. Finally, in the limiting case α=1, a rather monotonic curve is obtained in every non-local scenario, with a non-inverting sign of the group velocity.

Figure 5 shows dispersion relations and group velocities for several chains with different degrees of non-locality P for four significant values of α. As the value of α goes towards higher positive numbers, the behaviour of the monoatomic chain resembles the classical local discrete chain dispersion relation and the frequency increases, as in figure 5*a,b*. On the other hand, when α<−0.5, the stiffness of the springs connecting BNN masses increases, and the roton-like behaviour of the dispersion relation becomes more consistent, as the local minima in the curve become more evident (and thus the number of wavenumbers corresponding to a negative value of the group velocity increases), as in figure 5*g,h*. Figure 5*c–f* shows the transition for α=1 and α=−0.5, and those values are chosen to show how the dispersion curve loses the monotonic behaviour when the stiffness distribution parameter α gears towards negative values.

The frequency shift in the dynamical response is a promising feature, which allows for tuning of the stop bands in and the range of resonance frequencies; in the case of roton-like behaviour, the ω−κ relation is in fact not bijective, i.e. more than one κ values correspond to the same value of ω.

The increase in frequency for positive α values is tied to the overall stiffness increase when local links are favoured over non-local ones, as the overall stiffness is disadvantaged by the length of the connections.

### (e) Time domain simulations and sensitivity analyses

Information on the behaviour of an infinite chain undergoing mass-distribution modifications through changes in the parameter α can be retrieved through its dispersion relation. In this section, some insights on the behaviour of a finite mass-spring chain undergoing the same changes will be provided, so as to verify that the same kind of dispersion curves can be obtained from a finite model, taking into account some limitations. In fact, non-local models require an infinite number of elements in order for the non-locality level to be attained everywhere. As mentioned in §1, several methods are employed to reduce the boundary effects due to the finite nature of numerical and physical models. In this case—discrete—it is necessary for the ratio of the non-locality level P and the number of masses N to be under a certain threshold, as the bulk of the chain has to weigh more than the boundary in the dynamical behaviour. This is needed because the 2P boundary masses at the ends of the chain will not satisfy the non-locality conditions of the considered unit cell, only being connected on one side to their beyond nearest neighbours, and fixed on the other side. In order to keep the examples within a reasonable computational cost without losing any significant feature of the model nor suffering boundary effects, a 1000 masses chain with fixed ends is thus considered. The chain is excited by means of a harmonic force applied at the centre of the chain, with a chirp modulation entailing the resonance frequencies of the system.

The results were obtained from numerical simulations performed in Python through Newmark’s method [47]. The initial condition vectors were $\ddot{𝐔}=0$, $\dot{𝐔}=0$ and 𝐔=0, i.e. no initial acceleration, velocity or displacement were imposed on none of the masses. Mass and stiffness matrices for the finite system 𝐌,𝐊 have been assigned by taking into account the fixed ends. The system was then excited through a time-varying force on the central mass with a chirp modulation in frequency, in order to reconstruct the dispersion curves by acquiring the response to most of the eigenfrequencies of the finite system.

**Figure 6.**
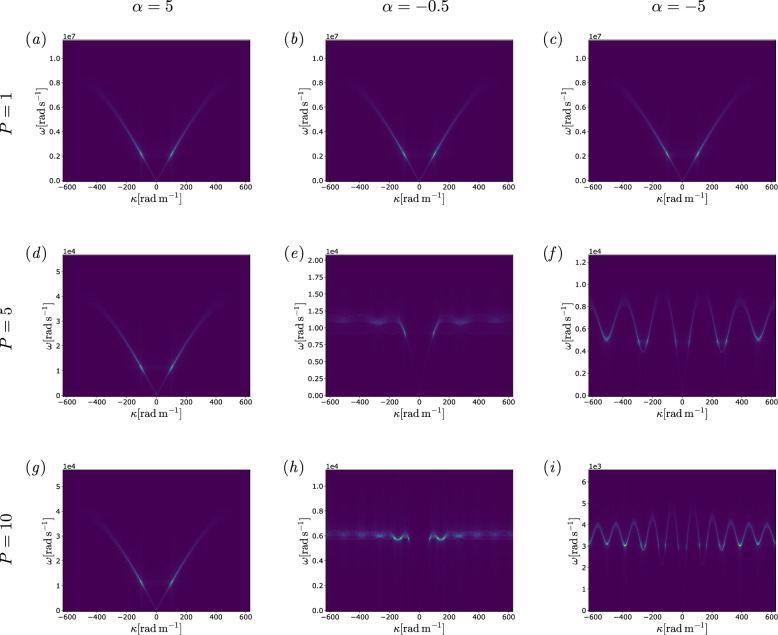
In this figure, (*a*–*i*) the A(κ,ω) contour plots for two-dimensional FFTs of the time-domain simulations are shown, where the amplitude A of the frequency response increases with the contrast between the dispersion curves and the background and depends on the excitation. In the graphs, the frequency ω in rad s^−1^ and the wavenumber κ in rad m^−1^ spanning the first Brillouin zone are respectively on the ordinate and abscissa axes. The chain is constrained at both ends, the masses being equal, with m=1kg, and the constants $E=500\;GPa,\;V=1\;m^{3},\;a=0.005\;m$. Input parameters entailed 1000 masses, level of non-locality P∈[1,10], input force $F=F_{0}cos(\omega (t)t)$, with $F_{0}=1\;N$ and forcing frequency ω varying at each time step through a chirp modulation, in the range of the natural frequencies of the chain. The images illustrate the propagation behaviour for different P and α values, as indicated explicitly on the left and top sides. Note that, despite growing levels of non-locality, when the value of α is positive no significant changes in the dispersion curves appear, as predicted by the theoretical model. On the contrary for negative values of α, the curves lose the monotonic behaviour and exhibit a number of local minima proportional to the non-locality level.

The time-domain results were then processed through a two-dimensional Fast Fourier Transform (FFT) to obtain the results in the frequency domain, i.e. the dispersion curves for the finite chain. The results can be observed in figure 6, where the numerical dispersion curves for mass-spring chains with levels of non-locality P=1,5,10 and stiffness distribution parameter α=−5,−0.5,5 are shown. The attained curves confirm the results predicted analytically for the infinite periodic chain, confirming the hypothesis that for a low enough ratio between the number of masses N and the level of non-locality P the boundaries do not interfere with the dynamical behaviour of the bulk of the chain. In figure 6*a–c* and the dispersion relations noticeably resemble the classical curves of a monoatomic local chain, regardless of the level of non-locality P, due to the positive value of the stiffness distribution parameter α. However, when the value of α reaches negative values, as in figure 6*d–i*, the roton-like effects can be retrieved, with the local minima and maxima being proportional to the level of non-locality of the chain.

## 3. Results and discussion

**Figure 7.**
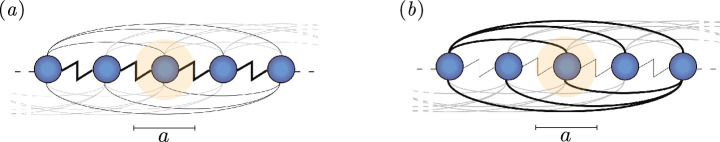
Comparison of local and non-local pairings with a positive (*a*) and negative (*b*) value of the stiffness distribution parameter α, which correspond respectively to stronger local (*a*) and nonlocal (*b*) pairings. The highlighted area corresponds to the periodic unit cell.

In this work, a mass-conservation law for the springs in a non-local monoatomic chain was conceived. Two parameters were then introduced, the non-locality level P and the stiffness distribution α, with the aim of allowing for tuneability in the models. The impact of the parameter α on how the mass and proportionally the stiffness distributes among the local and non-local pairings is summarized in the illustrations in figure 7, where in (*a*) and (*b*) α assumes, respectively, a positive and negative value.

Analytical predictions were made through infinite models, following Bloch–Floquet and Brillouin’s theories, and the role of the non-locality level P and the stiffness distribution parameter α in obtaining roton-like curves was highlighted. The obtained results were then validated through numerical simulations on a finite chain, which confirmed the analytical predictions.

Two main, equally relevant reasons motivated the choice of forcing the springs masses to be constant. One is a matter of mechanical consistency, which is believed to be required to any mono-atomic chain model comprising an arbitrary number of elastic links. In fact, when mass-spring models are employed to simulate dynamical systems, only masses are considered as contributors to the inertia and to the natural frequency of the structure. The usually local springs have negligible mass compared with that of the oscillating masses. However, as the non-local connections increase and the mass of the physical links increases accordingly, its contribution might become relevant to the dynamics, making this kind of model progressively less accurate as the level of non-locality (say the number of links) increases. In the limit of a ‘fully non-local’ chain made of infinite masses (i.e. all atoms are linked to each other), one would fall into the paradoxical situation of divergent total spring masses and the hypothesis of negligible mass of the links with respect to the mass of the atoms would be evidently violated. On the contrary, by imposing *a priori* a constraint on the total mass of the springs, the difference in the order of magnitude between the masses of the beads and of the connections would be automatically preserved, and thus the validity of the model. It is worth highlighting that forcing the springs mass to be constant is not a constraint to be reserved for one-dimensional non-local chain models, since the same line of reasoning holds true for two- and three-dimensional lattices. In addition, a note on non-local continua is perhaps due. In fact, for non-local continuous media (e.g. integral non-local elasticity, peridynamics and other models) the stiffness if modulated through the variation of the Young moduli with the distance between each pair of material points: as a consequence, at least in the absence of underlying hypotheses about the micro-structure, non-locality only depends on the intrinsic elasticity of the ground material and thus does not involve additional masses to be somehow balanced. The second reason for which it is felt that the conservation of springs mass must be considered as a necessary constraint to non-local models is related to a matter of engineering/physical rationality. In fact, it is well known that any problem in the structural mechanics context, be it static or dynamic, aimed at finding a design optimum, needs an object function and a constraint, the former furnishing the measure of the advantage one would gain in making a choice and the latter being exactly the parameter with respect one can compare two or more models. In Mechanics, for instance, design optimization of the shape of structures or topology optimization of materials are performed under prescribed boundary conditions and materials, selecting an object function (e.g. maximum stiffness) and imposing a constraint (e.g. the total mass of the element). In this way, two possible choices can be discriminated—and two models compared—by evaluating, among all the possible configurations (sizes of cross-sections in structurally optimized beams or spatially non-homogeneous density distribution in topologically optimized materials) the one(s) giving the best-needed performance, but under the given constraint (typically prescribing the total amount of available mass).

Concerning the applicability of the principle, some limitations need to be mentioned, as it is a theoretical one-dimensional model that describes a phenomenon to its bare main features. The applications to higher order models in two- or three-dimensional, for waves with transverse or composed components would require further study and taking into account more factors, as the overall geometry, local resonances or the axes for roton-like propagation. However, it is felt that even the limited information a one-dimensional model can give is crucial to the understanding of complex phenomena, when the simplification does not rule out important factors from the physics of the problem. A further limitation is in the number of non-local neighbours that can be connected in a finite model. In fact, when considering an infinite mass-spring chain, any number *P* of beyond nearest neighbour connections can be taken into account. On the other hand, when considering a finite model with *N* masses, the ends of the chains will be connected only on the left or on the right to the rest of the chain, thus supporting N−P non-local connections. The level of non-locality for which border effects can be ignored in the overall dynamical behaviour is thus proportional to the number of unit cells present in the designed model. Nevertheless, the study of the effects of the boundary conditions on the dynamics of non-local monoatomic chains would deserve a deeper reflection to analyse with due attention to their theoretical and physical implications, including sensitivity analyses to quantify the boundary influence over the behaviour of the bulk of the periodic system, which is beyond the purpose of the present work.

**Figure 8.**
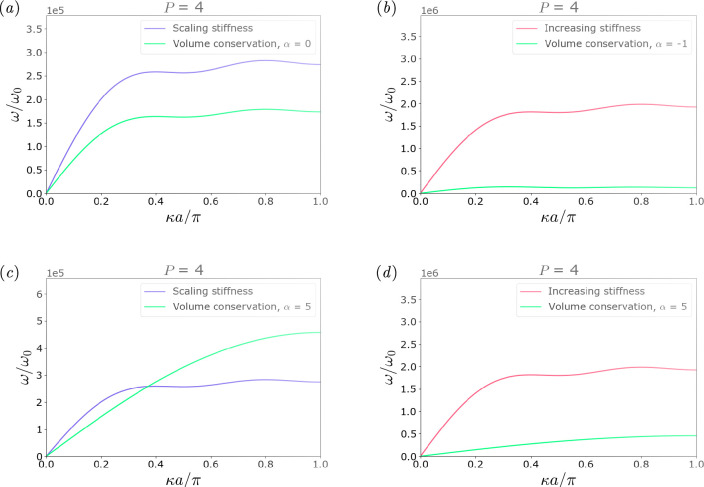
Comparison between the proposed volume-conservation model (in green), a model with stiffness scaling (decreasing) with distance (in blue, (*a*) and (*c*)) and a model with constant stiffness regardless of the level of non-locality, thus overall increasing with the number of pairings (in red, (*b*) and (*d*)), all three with a level of non-locality P=4. In (*a*) it can be noticed that for a chosen value of the α parameter a similar behaviour to the curve of the non-parametric model (in blue) can be obtained, as a similar scaling is attained in the volume-conserving case while retaining the mechanical constraint. The curve corresponding to the model presented in this work (in green) features a lower frequency range as the overall stiffness is affected by the volume conservation. The advantages of adopting a power law with a further degree of freedom in designing the non-local chain, given by α, are pointed out in (*c*), as by the sole tuning of the parameter the frequencies can be increased without switching to another scaling law. In (*b*) and (*d*), different behaviours retrieved by variation of α are compared to the fixed, non-tuneable, increasing stiffness model. The volume-preserving model allows for changes in the dispersion relation according to the selected α value within a frequency range which depends on the model's characteristics.


Some advantages for design purposes of the approach presented in this study are instead highlighted here. The volume, i.e. mass, conserving approach is tested in comparison to hypothetical scaling laws that one could employ to scale stiffness at different levels of non-locality and analyse non-local behaviour in mass-spring chains, which do not involve any mechanical consistency condition. In figure 8, some example dispersion relations are shown, with a direct comparison between the proposed model (in green) and two other models. The first of these models, shown in blue, is characterized by a stiffness with a scaling law in the form

(3.1) $$k_{j}^{\left(P\right)}=\frac{k_{1}^{\left(P\right)}}{j},\;\;\;j\in \{1,...,P\},$$

where


(3.2) $$k_{1}^{\left(P\right)}=\frac{EA_{0}}{a},$$

with $A_{0}$ and E constant. The stiffness is thus linearly decreasing with non-locality.

The second of these models, shown in red in the graphs, simply features the same stiffness for every spring added to the system, as


(3.3) $$k_{j}^{\left(P\right)}=k^{\left(P\right)}=C,\;\;\;\;\;\;\forall j\in \{1,...,P\},$$

with C being a given constant value, thus leading to an overall stiffening of the chain with the increase of non-local pairings and to a consequent rise in frequency. In the volume conservation hypothesis, the stiffness of each spring is constant in the case α=−1, however, the total volume (mass) remains constant and abides by the *mechanical consistency condition*.

It must be also pointed out that the model here proposed allows greater freedom in tuning the dynamic behaviour of a chain made by a fixed amount of material. By tuning the stiffness distribution parameter α, it appears clear how the proposed mass conservation law not only ensures mechanical consistency, but also allows us to span through multiple frequency ranges and behaviours that would need to be otherwise *a priori* fixed by the choice of a certain scaling law over another.

## 4. Conclusions and perspectives

**Figure 9.**
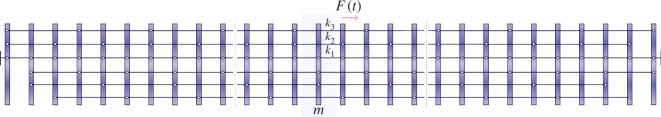
Possible scheme of a reduced and simplified experimental model, with fixed ends. The element marked by m represents the unit mass while the linear elements connecting them at different levels of non-locality are the linear elastic springs. The model is possibly hanged on a support, and free at both ends, so that the waves can completely reflect (or alternatively fixed at both ends to achieve the same effect). The number of unit cells present in the model should be enough to obtain a sufficient number of points in the space reciprocal to the coordinates space (x), that is the wavenumber space (κ). The model should be fixed in to limit the transverse modes as much as possible, as the first validation should be for a one-dimensional model as the imagined one. The dynamic excitation F(t) could be carried out by a waveform generator coupled with a piezoelectric patch on one end of the chain. The input signal would be designed through an iterative procedure to obtain the optimal frequencies, as it is necessary to excite as many natural frequencies as possible to construct the dispersion curves accurately. Furthermore, exciting higher frequencies could represent a challenge both in the exciting force and the retrieving of the displacement data. However, as in the case of the numerical simulation, the idea for the input signal would entail a chirp signal with frequencies $f=\omega_{i}/(2\pi )$, with $\omega_{i}\in \Omega =[0,\omega_{N}]$, where Ω is the set of the N eigenfrequencies of the N masses of the prototype (N≈100), that could be analytically or numerically estimated. The displacement amplitude u(x,t) for each mass could be subsequently retrieved for example by using high-speed cameras, subtracting the initial positions. The resulting data with an error estimate would then be treated by a Fourier transform to obtain the experimental dispersion curves.

Overall, the present proposal stems from the necessity and convenience of establishing a rule based on a clear physical meaning to determine the relationship between the degree of non-locality and the stiffness of the connections between the particles. The mass conservation is key here, not only for ensuring the mechanical consistency of the models, but also to allow fruitful dynamical comparisons between designs characterized by different parameter values. From a theoretical point of view, as stressed above, it is important for considering the springs massless in the mass-spring model that the dynamics are not influenced by the springs’ mass. The practical value of the principle answers to the need to compare different models for establishing the optimal level of non-locality and the way in which spring stiffnesses vary with the distance of the connected masses, with respect to an object function. Moreover, it is important for consistency of the comparisons between different dynamical models. As for further developments, an experimental validation of the proposed model could be envisaged. In figure 9, the scheme of a possible geometry is represented. Eventually, it would subsequently be possible to design mass conservative unit cells in more than one dimension and manufacture the prototypes with three-dimensional printing machines. However, as previously cited, it would entail more steps in the design process as choosing the kind of elastic waves, the most appropriate direction for the roton-like propagation in the imagined model and the possible interference given by local resonances.

Naturally, this line of reasoning can be easily extended to encompass more complex models, which may feature different interactions between masses, such as shear ones. For instance, the proposed conservation model would still apply in considering a classical shear stiffness of the type


(4.1) $$k_{j}^{\left(P\right)}=\frac{GA_{j}^{\left(P\right)}}{\chi ja},$$

where G is the shear modulus and χ the shear factor. If we were to consider bending stiffness or torsional stiffness, the expressions would instead read


(4.2) $$\begin{array}{rl} & k_{j}^{\left(P\right)}=\frac{12EI}{(ja{)}^{3}}\;\text{for bending,}\\ & k_{j}^{\left(P\right)}=\frac{GI_{p}}{ja}\;\text{for torsion,} \end{array}$$

where in the case of bending, a clamped configuration at both ends is considered. In these cases, the scaling on the cross-section $A_{j}^{\left(P\right)}$ is included in the inertia of the section, that could be considered for instance square with $I=\frac{A_{j}^{(P)2}}{12}$, or circular with $I_{p}=\frac{A_{j}^{(P)2}}{4\pi }$.

It is thus felt that the proposed mass conservation condition and the suggested criteria for guiding the transition from local to non-local monoatomic chains may help to conceive new classes of metamaterials and to design and realize three-dimensional prototypes, based for instance on advanced additive manufacturing processes.

## Data Availability

This article has no additional data.
